# Updated Surveillance Metrics and History of the COVID-19 Pandemic (2020-2023) in Sub-Saharan Africa: Longitudinal Trend Analysis

**DOI:** 10.2196/53409

**Published:** 2024-10-23

**Authors:** Alexander L Lundberg, Alan G Soetikno, Scott A Wu, Egon A Ozer, Sarah B Welch, Maryann Mason, Robert L Murphy, Claudia Hawkins, Yingxuan Liu, Charles B Moss, Robert J Havey, Chad J Achenbach, Lori A Post

**Affiliations:** 1 Buehler Center for Health Policy and Economics Robert J Havey, MD Institute for Global Health Northwestern University Chicago, IL United States; 2 Department of Emergency Medicine Feinberg School of Medicine Northwestern University Chicago, IL United States; 3 Feinberg School of Medicine Northwestern University Chicago, IL United States; 4 Department of Medicine, Division of Infectious Diseases Feinberg School of Medicine Northwestern University Chicago, IL United States; 5 Center for Pathogen Genomics and Microbial Evolution Robert J Havey, MD Institute for Global Health Northwestern University Chicago, IL United States; 6 Robert J Havey, MD Institute for Global Health Feinberg School of Medicine Northwestern University Chicago, IL United States; 7 Center for Global Communicable and Emerging Infectious Diseases Robert J Havey, MD Institute for Global Health Northwestern University Chicago, IL United States; 8 Institute of Food and Agricultural Sciences University of Florida Gainesville, FL United States; 9 Department of Medicine, General Internal Medicine and Geriatrics Feinberg School of Medicine Northwestern University Chicago, IL United States

**Keywords:** SARS-CoV-2, COVID-19, sub-Saharan Africa, pandemic, surveillance, public health, COVID-19 transmission, speed, acceleration, deceleration, jerk, dynamic panel, generalized method of moments, Arellano-Bond

## Abstract

**Background:**

This study updates the initial COVID-19 pandemic surveillance in sub-Saharan Africa (SSA) from 2020 by providing 2 additional years of data for the region.

**Objective:**

First, we aimed to measure whether there was an expansion or contraction in the pandemic in SSA when the World Health Organization (WHO) declared an end to the public health emergency for the COVID-19 pandemic on May 5, 2023. Second, we used dynamic and genomic surveillance methods to describe the history of the pandemic in the region and situate the window of the WHO declaration within the broader history. Third, we aimed to provide historical context for the course of the pandemic in SSA.

**Methods:**

In addition to updates of traditional surveillance data and dynamic panel estimates from the original study by Post et al (2021), this study used data on sequenced SARS-CoV-2 variants from the Global Initiative on Sharing All Influenza Data (GISAID) to identify the appearance and duration of variants of concern. We used Nextclade nomenclature to collect clade designations from sequences and used Pangolin nomenclature for lineage designations of SARS-CoV-2. Finally, we conducted a 1-sided *t*-test to assess whether regional weekly speed was greater than an outbreak threshold of 10. We ran the test iteratively with a rolling 6-month window of data across the sample period.

**Results:**

Speed for the region remained well below the outbreak threshold before and after the WHO declaration. Acceleration and jerk were also low and stable. The 7-day persistence coefficient remained somewhat large (1.11) and statistically significant. However, both shift parameters for the weeks around the WHO declaration were negative, meaning the clustering effect of new COVID-19 cases had become recently smaller. From November 2021 onward, Omicron was the predominant variant of concern in sequenced viral samples. The rolling *t*-test of speed equal to 10 was insignificant for the entire sample period.

**Conclusions:**

While COVID-19 continues to circulate in SSA, the region never reached outbreak status, and the weekly transmission rate remained below 1 case per 100,000 population for well over 1 year ahead of the WHO declaration. COVID-19 is endemic in the region and no longer reaches the threshold for its classification as a pandemic. Both standard and enhanced surveillance metrics confirm that the pandemic ended in SSA by the time the WHO made its declaration.

## Introduction

COVID-19, the disease caused by the SARS-CoV-2 virus, was first detected in Wuhan, China in the fall of 2019 [1-5]. The first confirmed COVID-19 case in sub-Saharan Africa (SSA) was reported in Nigeria on January 28, 2020 [6]. Our research team analyzed the pandemic in SSA 1 year into the pandemic [7]. This study provides 2 additional years of updated surveillance and analysis data for the region.

We have adopted the World Bank’s definition of SSA, which is based on economic development and geographical proximity, encompassing Angola, Benin, Botswana, Burkina Faso, Burundi, Cabo Verde, Cameroon, the Central African Republic, Chad, Comoros, the Democratic Republic of the Congo, Cote D’Ivoire, Equatorial Guinea, Eritrea, Eswatini, Ethiopia, Gabon, Gambia, Ghana, Guinea, Guinea-Bissau, Kenya, Lesotho, Liberia, Madagascar, Malawi, Mali, Mauritania, Mauritius, Mozambique, Namibia, Nigeria, Rwanda, Sao Tome and Principe, Senegal, Seychelles, Sierra Leone, Somalia, South Africa, South Sudan, Sudan, Tanzania, Togo, Uganda, Zambia, and Zimbabwe [8].

The World Health Organization (WHO) declared the end of the COVID-19 pandemic as a public health emergency of international concern on May 5, 2023 [9-11]. The decision was based on the recommendation of the COVID-19 Emergency Committee [11]. This study assessed whether the COVID-19 pandemic shifted to an endemic status in SSA at the time of the declaration.

Epidemiological terms, such as pandemic, epidemic, outbreak, and endemic, are used to describe the occurrence and spread of a disease [12,13]. The distinctions between these terms lie in their scope, geographical extent, and severity. An epidemic indicates a sudden increase in the number of disease cases in a specific population or region. If an epidemic spreads across several countries or continents, it becomes a pandemic. An outbreak, on the other hand, describes a sudden increase in a concentrated setting, usually involving a more limited geographical area than an epidemic. Endemic refers to the constant presence of a disease in a particular geographical region or population, with no sudden increases in case volumes [14,15].

Public health surveillance is the “ongoing, systematic collection, analysis, and interpretation of health-related data essential to planning and evaluation of public health practice” [16]. Surveillance not only explains the burden of death and disease but also generates research questions and guides researchers on topics that require further investigation [17-31]. Surveillance allows us to compare disease burden between geographical regions and to identify which regions are most impacted. This impact can be measured through the number of people contracting the disease, the number of people dying from the disease, and the associated costs.

Traditional surveillance carries several limitations that have been addressed by this study. Traditional surveillance provides a snapshot of what has already happened [17-31], meaning surveillance is static and focuses on the past. In the middle of a burgeoning pandemic, policymakers and public health practitioners also need to understand what is about to happen. Is an outbreak worsening? Will growth switch from linear to exponential? Are more people dying from a particular condition in one place than another? To inform health policy and practice, knowledge of what is about to happen is often more valuable than knowledge of what did happen. To that end, we have developed enhanced surveillance metrics that reflect the dynamics of a pandemic and inform imminent growth—most importantly, where along the epidemiological outbreak curve a particular region is situated. We have also included dynamic metrics about the speed of the pandemic at the national and regional levels. We have measured how the speed in this week compares to the speed in the last week, as well as how infections in 1 week predict infections in the next. We can think of the latter measure as the echoing forward of cases. These metrics have been tested and validated in prior research [7,32-42].

For this study, standard surveillance metrics explain what has already happened in SSA, while enhanced surveillance metrics explain what is about to happen or where along an epidemiological curve a country may sit. We have used both types of metrics to analyze the possible end to the pandemic in SSA.

This study has 3 objectives. First, we aim to measure whether there was an expansion or contraction in the pandemic in SSA when the WHO declared the end of the COVID-19 pandemic as a public health emergency of international concern on May 5, 2023. At both the region and country levels, we used advanced surveillance and analytical techniques to describe the status of the pandemic in a 2-week window around the WHO declaration. From a public health perspective, we need to know whether the rate of new COVID-19 cases is increasing, decreasing, or stable from week to week, and if any changes in the transmission rate indicate an acceleration or deceleration of the pandemic. Statistical insignificance is important as it can signal the epidemiological “end” to the pandemic if the rate of new cases is zero (or very low) and stable, meaning the number of new cases is neither accelerating nor decelerating.

Second, we used dynamic and genomic surveillance methods to describe the history of the pandemic in the region and situate the time window around the WHO declaration within the broader history. We considered the ratio of COVID-19 deaths to the number of transmissions as a proxy for the mortality risk from infection at the population level. We also included a historical record of genomic surveillance from sequenced viral specimens to identify the appearance and spread of variants of concern (VOCs) in the region.

Third, we aim to provide historical context for the course of the pandemic in SSA. We address several questions: How did countries respond to the pandemic? How did the region fare in terms of disease burden? What social, economic, and political factors shaped the course of COVID-19 in the region? This context can provide important lessons for disease prevention and mitigation in future pandemics.

## Methods

### Data Source

This study conducted trend analyses with longitudinal COVID-19 data from Our World in Data [43]. The study provides updates on traditional surveillance data and dynamic panel estimates from the original study by Post et al [7,40,41,44-46]. For the region of SSA, the data comprised an unbalanced panel of 47 countries and territories, running from December 11, 2020, to May 12, 2023. Because many countries around the world switched from daily to weekly reports at various points in 2023, we used a cubic spline to interpolate daily new cases and deaths if any country had 4 consecutive periods of nonzero new cases interspersed by 6 days of zero new cases.

To identify the appearance and duration of VOCs, we also used data on sequenced SARS-CoV-2 specimens from the Global Initiative on Sharing All Influenza Data (GISAID) [47-51]. We used Nextclade nomenclature [52] to collect clade designations from sequences and Pangolin nomenclature for lineage designations of SARS-CoV-2 [53,54]. Metadata for the study period were collected on June 22, 2023. To avoid low frequency or potentially erroneous samples, the data were filtered to exclude months with fewer than 100 available samples, variant groups with fewer than 5 samples in a month, and variant groups representing less than 0.5% of the total samples in a month. The final data set consisted of 184,386 total samples available on the GISAID platform [48-51].

### Measures

This study employed the traditional surveillance metric, “speed” of spread, defined as the rate of new COVID-19 cases per 100,000 population. Additional enhanced metrics include acceleration, jerk, and 1- and 7-day persistence metrics. “Acceleration” is the change in speed from 1 unit of time to the next. Acceleration identifies whether the speed of spread is rising (positive acceleration), falling (negative), or stable (zero). “Jerk” is the change in acceleration over time. Its name comes from physics nomenclature. A large positive jerk can signal explosive growth in transmission rates. The 1- and 7-day persistence metrics are the statistical impacts of the 1- and 7-day lags of speed on the current speed. These measures capture an echo-forward effect of COVID-19 cases on cases either 1 or 7 days later. They are coefficient estimates from an Arellano-Bond dynamic panel data model [55]:

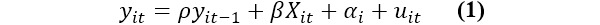



The dependent variable is speed, and the independent variables include weekend and recent week indicators. Weekend indicators account for changes in data reports across weekends, and recent week indicators account for changes in the status of the pandemic in the most recent 2 weeks, which allows for an assessment of stability in the weeks before and after the WHO declaration. Lastly, *α_i_* is a country fixed effect, which controls for time-invariant country-specific factors, and *u_it_* is the idiosyncratic error term. See the initial study for details [7]. The persistence metrics are calculated on a rolling 120-day window to limit biases caused by changes in testing capacity and other factors over time.

We also analyzed the potential “statistical end” to the pandemic with a 1-sided *t*-test for whether the mean speed was equal to or greater than the outbreak threshold of 10. We ran the test on a rolling 6-month window of weekly speed for the region, and we plotted the *P* values from the test over time. All statistical analyses were conducted in R (version 4.2.1; R Project for Statistical Computing) with the *plm* package (version 2.6-2) [44,45].

### Ethical Considerations

The data in this study are publicly available and contain no identifiable or private information. As defined by the 45CFR46:102 policy, the study does not qualify as human subjects research. Sources have been presented in the Data Availability statement.

## Results

Table 1 presents the dynamic panel estimates for the most recent time window. The Wald test for the regression was significant (χ^2^_6_=27947.46; *P*<.001), and the Sargan test failed to reject the validity of the overidentification restrictions (χ^2^_540_=45; *P*>.99). While the 1-day lag coefficient was approximately zero and statistically insignificant, the 7-day lag coefficient was much larger and significant (1.106), suggesting a cluster effect in which 1 case on a given day predicts 1.106 cases 7 days later. The shift parameters for both weeks around the WHO declaration were negative (but insignificant), suggesting that the clustering effect had recently become smaller.

**Table 1:** Arellano-Bond dynamic panel data modeling of the number of daily infections reported by country in sub-Saharan Africa (April 28 to May 12, 2023).

Variable	Value	*P* value
1-day persistence coefficient	–0.000	.99
7-day persistence coefficient	1.106	<.001
Shift parameter week of May 1	–0.227	.07
Shift parameter week of May 8	–0.044	.58
Weekend	–0.037	.12

The dynamic panel model is motivated by the limitations of the basic reproductive number, R_0_, which is the average number of people 1 contagious person will infect [56]. While R_0_ is a useful statistic, it depends on many factors, such as public health policy, vaccination rates, demographics, and the transmissibility of a pathogen. The SARS-CoV-2 virus mutated many times over the sample period. When coupled with the evolution of public health policy and other factors shaping R_0_, this reality makes continually updated estimates of R_0_ difficult to obtain. The dynamic panel estimates are calculated on a rolling 120-day window, so they can adjust rapidly over time. Finally, the Arellano-Bond model is robust to time-invariant unobservable factors (any stable differences between countries over time), corrects for autocorrelation, and allows for statistical tests of the model parameters [41].

The Wald and Sargan tests are traditionally used to examine the validity of the Arellano-Bond dynamic panel model. Under the null hypothesis in the Wald test, the independent variables collectively have no power to explain movements in the dependent variable. The test statistic was highly significant (*P*<.001), implying that the independent variables do have explanatory power. Under the null hypothesis of the Sargan test, in contrast, the overidentifying restrictions assumed in the estimation of the model are valid. A rejection of the null hypothesis would therefore be evidence against the validity, but the test failed to reject the null hypothesis with a test statistic *P* value approaching 1.

Static surveillance metrics for the week of April 28 are provided in Table 2. Analogous metrics for the week of May 5, 2023, are presented in Multimedia Appendix 1. Every country had a small number of new COVID-19 cases. The highest number of cases was seen in Mauritius for the week of April 28 (n=229), which had a transmission rate of 18 new cases per 100,000 population. Outside of Mauritius, the next highest transmission rate was seen in Cabo Verde the next week (4.57). While Mauritius was in an outbreak, the latter transmission rate is considered a low transmission rate by the Centers for Disease Control (CDC) [7,32-42,57]. Specifically, “low” transmission is defined by no more than 10 cases per 100,000 people per week, “moderate” transmission is 10 to 50 cases per 100,000 people per week, and “substantial” transmission is 50 to 100 cases per 100,000 people per week [57,58].

The status of the pandemic around the WHO declaration in SSA is consistent with an “end” to the pandemic. Only Mauritius was in a state of a moderate outbreak, and island nations often vacillate between high and low transmission rates. Thus, COVID-19 could be considered an epidemic in Mauritius but endemic in the region. However, we do note that the high number of countries with zero new COVID-19 cases may reflect a regime change in data reporting, with fewer countries reporting data than earlier in the pandemic.

Table 3 contains enhanced dynamic surveillance metrics for the week of April 28, 2023. As before, similar metrics for the week of May 5, 2023, are available in Multimedia Appendix 2. Speed was low for every country, except for Mauritius. Because only a single country was in an outbreak, epidemiologically, COVID-19 could be considered an epidemic in Mauritius but did not reach the threshold of a pandemic. Islands are known to experience more volatility in transmission rates [59,60].

**Table 2:** Traditional COVID-19 surveillance metrics for countries in sub-Saharan Africa for the week of April 28, 2023.

Country	New COVID-19 cases, n	Cumulative COVID-19 cases, n	7-day moving average of new cases	Infection rate per 100,000 individuals	New deaths, n	Cumulative deaths, n	7-day moving average of deaths	Death rate per 100,000 individuals	Conditional death rate
Angola	0	105,384	0	0	0	1934	0	0	0.02
Benin	0	28,014	0	0	0	163	0	0	0.01
Botswana	1	329,856	1	0.18	0	2796	0	0.04	0.01
Burkina Faso	0	22,056	0	0	0	396	0	0	0.02
Burundi	0	53,740	1.29	0	0	15	0	0	0.00
Cabo Verde	25	63,599	18.86	4.21	0	413	0	0	0.01
Cameroon	0	125,036	0.71	0	0	1972	0	0	0.02
Central African Republic	0	15,367	0	0	0	113	0	0	0.01
Chad	0	7698	0	0	0	194	0	0	0.03
Comoros	0	9109	0	0	0	160	0	0	0.02
Côte d'Ivoire	1	88,326	0.43	0	0	834	0	0	0.01
Democratic Republic of Congo	32	96,403	28.57	0.23	0	1465	0.14	0	0.02
Equatorial Guinea	0	17,130	0	0	0	183	0	0	0.01
Ethiopia	2	500,847	3.71	0	0	7574	0	0	0.02
Gabon	0	48,992	0	0	0	307	0	0	0.01
Gambia	0	12,626	0	0	0	372	0	0	0.03
Ghana	0	171,653	0	0	0	1462	0	0	0.01
Guinea	0	38,563	0	0	0	468	0	0	0.01
Guinea-Bissau	0	9614	0	0	0	177	0	0	0.02
Kenya	1	343,073	0.29	0.01	0	5688	0	0	0.02
Liberia	0	8090	0	0	0	294	0	0	0.04
Madagascar	3	68,236	3.14	0.06	0	1424	0	0	0.02
Malawi	1	88,638	1	0	0	2686	0	0	0.03
Mali	0	33,144	0	0	0	743	0	0	0.02
Mauritania	4	63,653	4.57	0	0	997	0	0	0.02
Mauritius	229	302,695	227.86	18.09	0	1048	0.29	0	0.00
Mozambique	0	233,417	0	0	0	2243	0	0	0.01
Namibia	0	171,310	0	0	0	4091	0	0	0.02
Niger	0	9513	0	0	0	315	0	0	0.03
Nigeria	0	266,675	0	0	0	3155	0	0	0.01
Republic of the Congo	0	25,192	0.43	0	0	389	0	0	0.02
Rwanda	0	133,194	0	0	0	1468	0	0	0.01
São Tomé and Príncipe	0	6569	0	0	0	80	0	0	0.01
Senegal	0	88,997	0.29	0	0	1971	0	0	0.02
Seychelles	0	50,937	0	0	0	172	0	0	0.00
Sierra Leone	0	7762	0	0	0	125	0	0	0.02
Somalia	0	27,334	0	0	0	1361	0	0	0.05
South Africa	0	4,072,533	0	0	0	102,595	0	0	0.03
Sudan	0	63,993	0	0	0	5046	0	0	0.08
Swaziland	0	74,670	1.86	0	0	1425	0	0	0.02
Tanzania	0	43,078	0.57	0	0	846	0	0	0.02
Togo	0	39,487	0.57	0	0	290	0	0	0.01
Uganda	13	170,671	11	0	0	3632	0	0	0.02
Zambia	0	343,911	0	0	0	4058	0	0	0.01
Zimbabwe	2	264,719	1.29	0.07	0	5688	0.43	0	0.02

**Table 3:** Novel surveillance metrics for countries in sub-Saharan Africa for the week of April 28, 2023.

Country	Speed	Acceleration	Jerk	7-day persistence effect on speed
Angola	0	0	0	0
Benin	0	0	0	0
Botswana	0.16	0	0	0.19
Burkina Faso	0	0	0	0
Burundi	0.01	0	0	0.01
Cabo Verde	3.18	0.60	0.89	2.98
Cameroon	0.02	0	0	0.08
Central African Republic	0	0	0	0
Chad	0	0	0	0
Comoros	0	–0.07	–0.07	0.07
Côte d'Ivoire	0	0	0	0
Democratic Republic of Congo	0.20	0.01	0	0.16
Equatorial Guinea	0	0	0	0
Ethiopia	0	0	0	0
Gabon	0	0	0	0
Gambia	0	0	0	0
Ghana	0	0	0	0.02
Guinea	0	0	0	0.04
Guinea-Bissau	0	0	0	0
Kenya	0	0	0	0.01
Liberia	0	0	0	0
Madagascar	0.07	0	0	0.10
Malawi	0	0	0	0
Mali	0	0	0	0
Mauritania	0.11	0	0	0.03
Mauritius	17.58	0	0	15.76
Mozambique	0	0	0	0.03
Namibia	0	0	0	1.33
Niger	0	0	0	0
Nigeria	0	0	0	0
Republic of the Congo	0	–0.01	–0.01	0.01
Rwanda	0	0	0	0
São Tomé and Príncipe	0.06	0	0	0.41
Senegal	0	0	0	0
Seychelles	0	0	0	0
Sierra Leone	0	0	0	0
Somalia	0	0	0	0
South Africa	0	0	0	0
Sudan	0	0	0	0
Swaziland	0.51	0	0	0.53
Tanzania	0.01	–0.01	0	0.04
Togo	0.01	0	0	0.02
Uganda	0.02	0	0	0.02
Zambia	0	0	–0.01	0.40
Zimbabwe	0.05	0	–0.01	0.03

**Figure 1:**
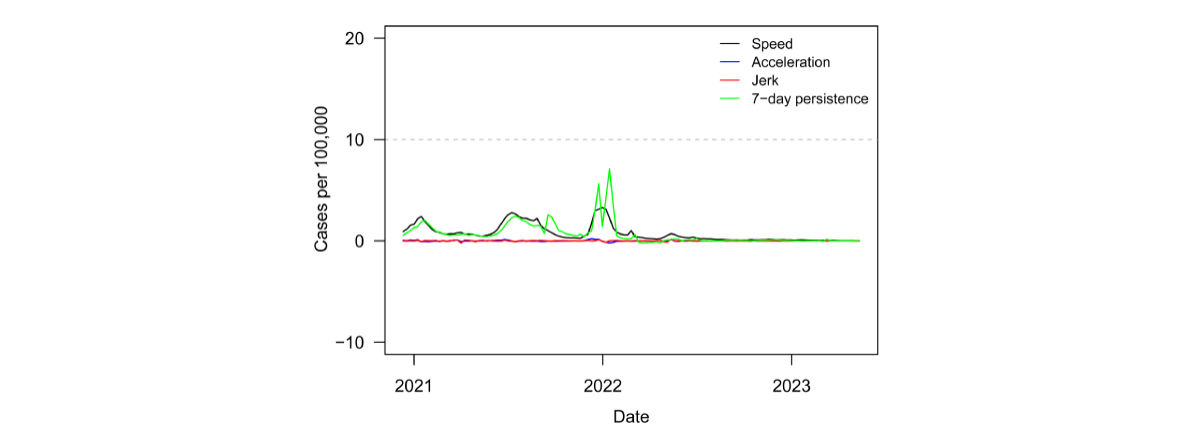
Novel surveillance metrics (speed, acceleration, jerk, and 7-day persistence) for COVID-19 infections in sub-Saharan Africa from December 2020 to May 2023.

The conclusion of the transition of COVID-19 from “pandemic” to “endemic” might be premature. If acceleration and jerk values are large in magnitude, low transmission rates would not be stable. However, acceleration and jerk values were zero or near zero for every country. Low data reporting may explain part of this stability. Figure 1 provides a longer timeline, which shows that the stability was not a recent feature but had been present for months up to the WHO declaration. Finally, the 7-day persistence effect on speed was also low, zero, or negative, suggesting little propagation of cases in the week before the current week. These metrics together suggest that the pandemic might have indeed ended for the region. It is important to note that the values in Table 2 and Multimedia Appendix 1 are not calculated as day-over-day averages across the week, as they are in Table 3 and Multimedia Appendix 2. Thus, the magnitudes of speed may not exactly match across tables.

Figure 1 plots regional speed, acceleration, jerk, and 7-day persistence metrics from December 11, 2020, to May 12, 2023. Descriptions of each measure are presented in the Methods section. The region never reached the outbreak threshold. In fact, the highest speed ever reached was 3.3. Furthermore, speed remained below 1 since the end of January 2022. Relative to other global regions, SSA was relatively unscathed by the pandemic. For example, North America, Europe, East Asia, and the Pacific saw maximum speeds eclipse 200 in outbreaks driven by the Omicron variant.

**Figure 2:**
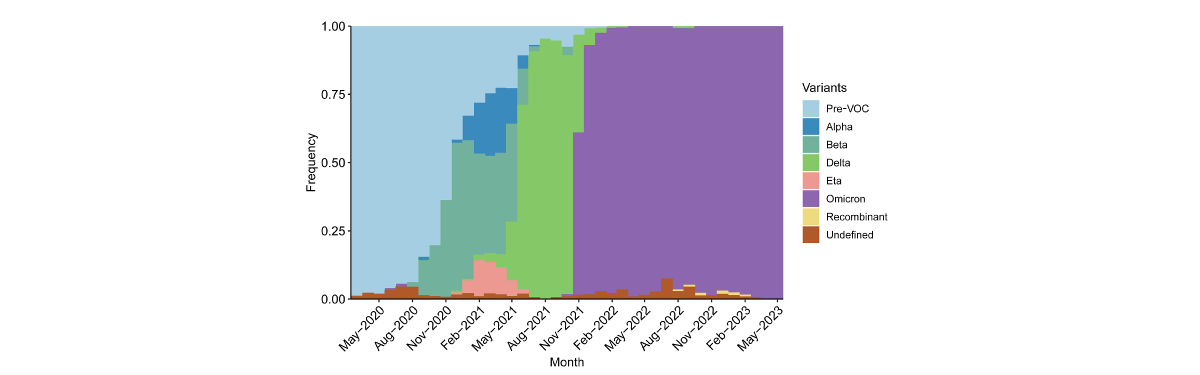
Variants of concern (VOCs) as a proportion of all sequenced SARS-CoV-2 specimens from March 2020 to May 2023 in sub-Saharan Africa (N=126,638).

Figure 2 plots variant groups as a proportion of all viral specimens collected and sequenced in the region (and made available through GISAID) each month. Interestingly, the region did not experience a large outbreak driven by the Omicron variant despite its discovery in Botswana and South Africa. Much of the rest of the world saw a surge in cases amid the heightened transmissibility of Omicron [61]. Another potential indication of the end of the pandemic is the continued dominance of the Omicron variant. While the region saw a mixture of the ancestral, Alpha, Beta, Delta, and Eta variants prior to the arrival of Omicron in November 2021, viral sequences almost exclusively indicated the Omicron variant and its subvariants ever since. The proportion of all specimens being identified as the Omicron variant (indicated by purple in Figure 2) was nearly 100% since the time the Omicron variant first appeared.

**Figure 3:**
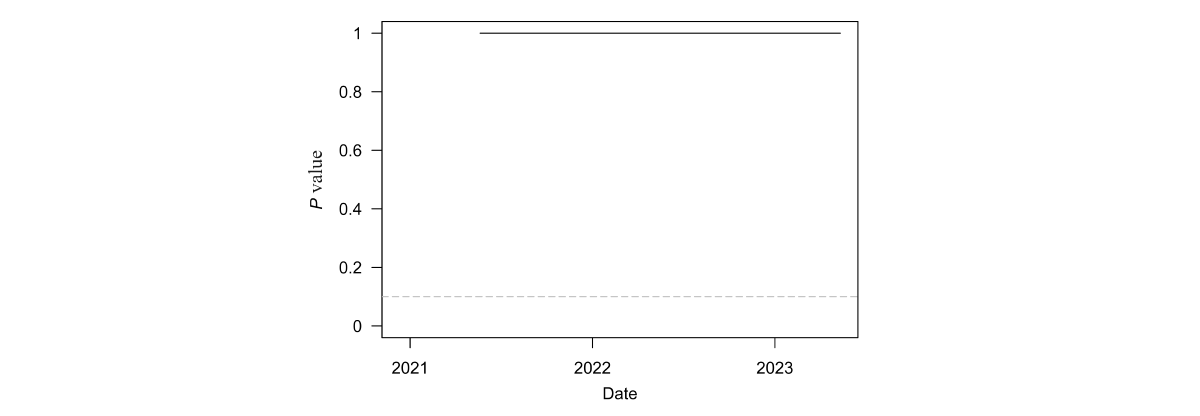
*P* values from t-tests of weekly COVID-19 transmission per 100,000 population equal to 10 over a rolling 6-month window in sub-Saharan Africa.

Figure 3 plots *P* values from a series of 1-sided *t*-tests of whether speed for the region was equal to or greater than the outbreak threshold of 10. These tests were conducted on a rolling 6-month window of weekly regional speed. The dashed grey line denotes the least restrictive conventional significance level threshold of α=.10. The test was insignificant for the entire sample period. This lack of statistical significance is consistent with the end of the pandemic in the region, as the test clearly failed to reject the null hypothesis of outbreak threshold speed at any point in time.

**Figure 4:**
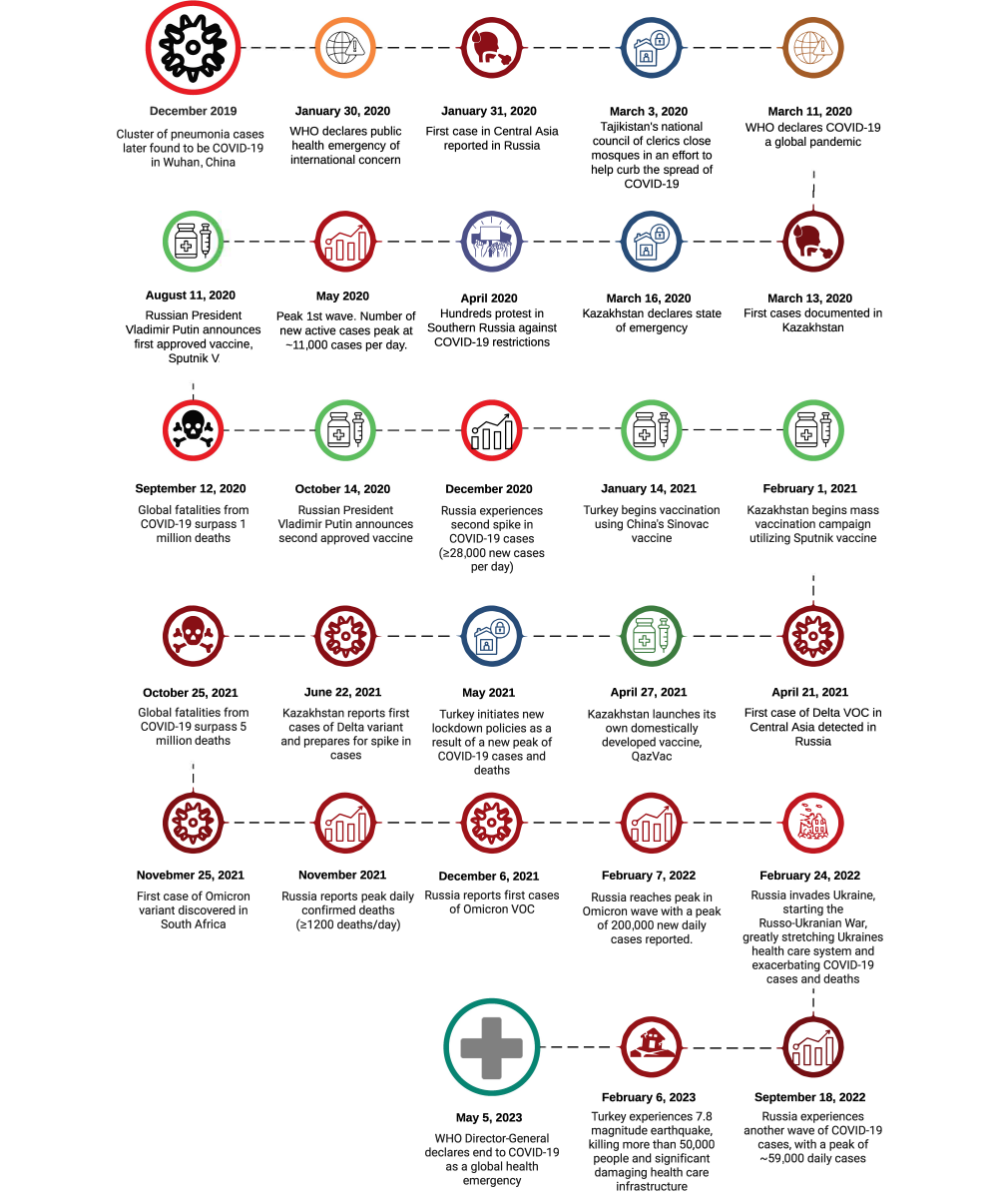
Timeline of the COVID-19 pandemic in sub-Saharan Africa. CDC: Centers for Disease Control; SSA: sub-Saharan Africa; VOC: variant of concern; WHO: World Health Organization.

With the historical context of enhanced surveillance metrics, the region appears to be at the end stage of the pandemic. Figure 4 provides a timeline of the onset of COVID-19 in SSA as well as vaccination programs and policies that shaped the course of the pandemic in the region.

## Discussion

### Principal Findings

The first objective of this study was to describe the status of the COVID-19 pandemic in SSA up to the point when WHO declared an end to COVID-19 as a public health emergency. The weekly transmission rate remained below 1 case per 100,000 population for over a year. Both dynamic and genomic surveillance methods suggested that COVID-19 transitioned from “pandemic” to “endemic” in SSA. For historical context, the overall region never reached the status of an outbreak. In general, SSA faced lower caseloads and fatalities from COVID-19 than the rest of the world. For example, during the first wave of the pandemic in early 2020, SSA as a region had significantly lower cases and deaths compared to individual countries such as Germany, Italy, and the United States. By early 2021, the region reported 3.2 million cumulative COVID-19 cases and 83,000 deaths, which accounted for only 2% of global cases and deaths despite the region hosting over 1.1 billion people or 14% of the global population [62].

One possible explanation for the low disease burden is undercounting driven by a lack of strong health care infrastructure in SSA [62-64]. The region went into the pandemic with limited health care facilities, personal protective equipment (PPE), and health care providers [65]. For example, in 2020, countries in SSA on average had 1.8 hospital beds per 1000 people compared to 4.41 hospital beds per 1000 people in Europe [66-68]. Some countries have also faced years of political instability. For example, South Sudan has dealt with nearly a decade of armed conflict, which has weakened the health care system and limited the ability to track outbreaks.

However, testing capacity in the region quickly expanded from a somewhat strong foundation, as the region had grappled with infectious diseases, such as malaria and Ebola, for years ahead of the COVID-19 pandemic [69,70]. Furthermore, estimates from several studies indicate that Africa had an undercounted death rate in line with the rest of the world [71-73]. The number of tests per COVID-19 case was also similar or higher in many African countries than in other countries around the world, even early in the pandemic [71].

The 47 countries in SSA had fewer than 0.2 million cumulative deaths from COVID-19. At the time of writing this article in June 2024, the entire continent had approximately 250,000 deaths, according to Our World in Data [43]. In comparison, South America had 1.4 million deaths, North America had 1.7 million deaths, Europe had 2.1 million deaths, and Asia had 1.6 million deaths. The rounded population sizes of Africa, South America, North America, Europe, and Asia were 3.9 billion, 0.4 billion, 0.7 billion, 0.6 billion, and 4.7 billion, respectively. Without dramatic differences in death undercounts, other explanations must account for part of the relative performance of Africa.

If testing cannot explain such a large gap, another potential explanation for the low death rate in the region is its unique demographic and geographic profile. SSA has a significantly younger population than other areas of the world [64]. For example, individuals older than 65 years accounted for 16% of the United States population but 3% of the SSA population in 2020 [74]. COVID-19 is known to disproportionately affect the health of elderly people, and demographic differences may explain why some developed countries faced significantly higher rates of mortality compared with SSA [63]. Geographically, most of the region’s population (59%) lives in rural areas, and this proportion is greater than that in other global regions [64].

Given that the region hosts a variety of communicable diseases, including malaria, typhoid, and Ebola, it is also plausible that repeated exposures to various pathogens helped people in SSA build stronger immune systems that rendered them less vulnerable to COVID-19 [75]. Still, COVID-19 had long-term health and societal impacts in the region. Given the limited financial resources of many countries in SSA, prolonged containment strategies became unsustainable as millions of people were thrown into poverty [76]. The Delta variant, which was originally sequenced in India, became the dominant strain in the region by the middle of 2021 and contributed to a third wave of infections [77]. The first cases of the Omicron variant were discovered in Botswana and South Africa in November 2021. Immediately after the discovery, countries across the globe implemented travel bans to countries in SSA to stem potential viral spread. However, these efforts did not stop Omicron from spreading globally and led to negative socioeconomic impacts in the region [78].

The SSA economy was greatly affected by the pandemic. During 2020, the region experienced a 2.1% gross domestic product (GDP) contraction, which is largely attributable to decreased economic activity from COVID-19 restrictions [79]. Lockdowns disrupt daily life and potentially limit access to nutrition, education, and health services. The African Development Bank estimated that about 30 million people in the region were pushed into extreme poverty as a result of the pandemic [80]. Lockdowns had an especially large effect on urban centers where, due to high population density, governments used strict lockdown protocols to control the spread of the virus. Additionally, quarantine measures disrupted food supply chains and threatened food security for millions of people in SSA [81]. It is estimated that 19.3% of the population could no longer afford their prepandemic level of food consumption due to economic hardships brought on by lockdown measures [82].

In general, governments used economic packages to cushion the financial blow on individuals and businesses. These packages typically included direct cash transfers, tax relief, investment in health care infrastructure, and investment in specifically vulnerable sectors of the economy. For example, Nigeria announced on April 1, 2020, that it would make cash transfers of 20,000 Naira (US $52) to low-income households registered in the government’s National Social Register. On April 21, 2020, South Africa announced a massive US $26 billion (10% of GDP) stimulus package, most of which was allocated to health expenditure, wage protection through the Unemployment Insurance Fund, and financial support for small businesses [83]. Many SSA countries also implemented indirect forms of economic support. For example, Ghana provided all citizens with free water from April 2020 to June 2020 [84]. Some governments also took steps to ensure food security by providing food rations to vulnerable populations [85]. After the initial lockdown in 2020, Rwanda promptly began distributing several food staples, including rice, beans, flour, and cooking oil, to vulnerable households [86]. These supplies were delivered “door to door” in an effort to maintain social distancing and thus minimize the spread of the virus.

International financial organizations also directed approximately US $230 billion during 2020 and 2021 to help countries in SSA mitigate the impact of the pandemic. For example, in April 2020, the African Development Bank Group created a US $10 billion COVID-19 Rapid Response Facility and a US $3 billion “Fight COVID-19 Social Bond” to help combat fiscal challenges [87].

### Policies Implemented to Control and Mitigate the Transmission of COVID-19

SSA has several endemic diseases, such as Ebola, tuberculosis, HIV, and malaria, which have provided countries with valuable experience in how to combat disease outbreaks. For example, the African Union formed the Africa CDC in 2017 as a direct response to the 2014-2016 Ebola outbreaks. On February 22, 2020, the Africa CDC, in conjunction with the Southern Africa Center for Infectious Disease Surveillance (SACIDS), formed the Africa Taskforce for Coronavirus Preparedness and Response (AFTCOR) to create a continent-wide strategy for COVID-19 and bolster surveillance efforts in high-risk countries [88]. Surveillance efforts were built on existing systems in place for monitoring influenza and other severe acute respiratory diseases. Individual governmental responses in SSA involved travel bans, social distancing measures, public masking, contact tracing, and widespread lockdowns. Forty-four SSA countries closed schools, banned public gatherings, and enacted social distancing measures. Moreover, 11 SSA countries declared a state of emergency, and 15 countries closed their air, sea, and land borders [89]. South Africa, which had the region’s highest reported cases and deaths, was one of the first countries to implement strict lockdown measures. Within days of its first confirmed case, South Africa declared a national state of disaster [90]. By March 26, 2020, the government imposed a 21-day national lockdown, which restricted all nonessential movement of people and goods [91]. The government also used mobile testing units to reduce the movement of potentially infected individuals through communities [92]. Nigeria’s initial COVID-19 strategy included a nationwide curfew, public masking, and a ban on interstate travel. However, the country quickly shifted to a targeted lockdown strategy in specific areas with rapidly increasing COVID-19 cases. Kenya implemented a partial lockdown, with the government imposing a dusk-to-dawn curfew and restricting travel in and out of the capital, Nairobi, and other COVID-19 hotspots [93]. Uganda closed schools for almost 2 years to combat the pandemic, which is the longest school closure in the world [94].

Given the shortages of PPE and other essential medical equipment, such as ventilators, many countries shifted manufacturing to fill their gaps. A factory in Kenya was rapidly transformed from manufacturing clothes to producing 30,000 surgical masks a day [95]. The Central African Republic helped contract and support over 18,000 tailors and 300 firms by having them create protective face masks. This project not only bridged the gap in their PPE shortage but is estimated to have injected US $17 million into the economy [96]. South African manufacturers shifted tasks to address national shortages in ventilators [97].

Ultimately, countries focused on achieving herd immunity through widespread vaccination campaigns. However, the region faced difficulties accessing the limited global COVID-19 vaccine stockpiles, especially low-income countries [98]. It was estimated that US $12.5 billion would be required to vaccinate 70% of the SSA population and achieve herd immunity. This cost would equate to US $15.17 per person in the region, which is approximately 150% of the annual per capita governmental health care expenditure in the region [99]. As a result, many countries turned to international consortiums to fill the financial gap. For example, the African Vaccine Acquisition Team of the African Union and COVAX, a WHO-led consortium designed to ensure equitable access to vaccines, sought to acquire millions of vaccine doses [100]. However, vaccinations in SSA substantially lagged behind the rest of the world. By late 2021, when over 70% of high-income countries had over 40% of their populations vaccinated, Africa had only vaccinated 6% of its population [101]. As of March 24, 2023, just over 3 years after the start of the COVID-19 pandemic, less than 30% of the population of SSA had been fully vaccinated [102].

One factor contributing to low vaccination rates was vaccine hesitancy due to a perceived lack of safety and efficacy [103]. Another limiting factor was the lack of resources and infrastructure in certain countries to effectively distribute the vaccine. For example, one report on public health care in SSA showed that 1 in 6 people in the region lived more than 2 hours away from the closest public hospital [104]. Governments ultimately turned to mass vaccination campaigns focused on community engagement and education. For example, Cameroon, which had one of the lowest COVID-19 vaccination rates as of November 2022, implemented a 10-day mass vaccination campaign that ultimately administered over 2 million doses, almost doubling the percentage of the population that had received at least one dose [105]. The campaign focused on mobilizing community leaders to disseminate accurate information regarding the effectiveness of the vaccines [105]. The Central African Republic carried out a similar 7-day mass vaccination campaign against both COVID-19 and tetanus during November 2022 [105].

Despite success in ramping up testing capacity in the region, economic constraints pose challenges for future pandemic readiness [106,107]. Regional cooperation is likely to be a necessity [106]. Meta-analyses suggest that population knowledge may have been an impediment to the use of PPE and other mitigation measures at the individual level [108]. A combination of economic resources to secure vaccines, coupled with information campaigns, may encourage the future uptake of vaccines, which has remained low in SSA as a whole over the course of the COVID-19 pandemic [102]. Still, while the region grappled with economic hardship from the pandemic, the disease burden in SSA was among the lowest in the world.

### Limitations

COVID-19 data became less frequently reported around the world by the time the WHO declared an end to the pandemic as a public health emergency [109]. The number of countries with zero reported cases around the time of the declaration suggests a regime change in data reports for the region (as well as for other global regions). Additionally, people began to use at-home tests as the pandemic evolved, though this change was less pronounced in SSA than in other parts of the world due to low rates of at-home tests [106,110]. Because the surveillance metrics of speed, acceleration, jerk, and 7-day persistence are based on rates and not total counts, statistical bias in regional estimates caused by countries moving in or out of the sample has been mitigated, but the omission of a country could still influence historical data comparisons. The persistence measures from dynamic panel estimates have been calculated from a 120-day window to limit intertemporal biases caused by changes in reporting or sample inclusion. Still, data availability remains a limitation. Continued reports on COVID-19 transmission after the middle of May 2023 would provide further insights into the status of the pandemic at the time. The team did not consider transmission data to be sufficient for conclusions beyond that point.

### Conclusion

Although COVID-19 is now well contained in SSA, concerns about the potential resurgence of the virus remain valid. As long as COVID-19 continues to spread and mutate, the possibility of novel variants remains. Variants could potentially be more transmissible or resistant to vaccines, or cause more severe illness. This underlines the importance of continued vigilance, vaccination efforts, and global cooperation to control the spread of the virus [39].

SSA experienced a relatively low COVID-19 disease burden compared to other global regions, such as the Americas and Europe [43]. This relative performance may seem counterintuitive given the economic and health infrastructure disparities across regions, as well as the relatively low vaccination rates across SSA. However, SSA has several distinctive and protective factors. Demographics are the primary protective factors. The African continent as a whole has the youngest population in the world, and COVID-19 mortality rates rise exponentially with age [111-113]. Moreover, international trade and air pollution have been positively linked to transmission rates [114,115]. International trade between SSA countries remains stubbornly low [116], but as the region continues to develop economically, the risk of novel pathogen transmission through trade routes may grow. The climate may also be protective, but the relationship between climate and COVID-19 transmission is complex, and risk profiles will shift as climate change continues [117,118].

Several lessons for future pandemic preparedness have emerged. Perhaps the most pressing question is how to limit disease burden ahead of vaccines and treatment modalities. New technologies will renew the potential to improve early surveillance methods [119-121]. A combination of rapid widespread individual testing, together with epidemiological task forces, should be the first line of defense [122]. Lockdown policies, despite their economic cost, have also shown efficacy [123]. When vaccines do become available, measures of general governance are associated with vaccination rates, and individual countries with low general governance measures may be points of intervention to contain disease spread [124]. On that note, continued international cooperation will be necessary to mitigate the impact of future pandemics both inside and outside SSA [125,126].

## Appendix

Multimedia Appendix 1 Traditional COVID-19 surveillance metrics for countries in sub-Saharan Africa for the week of May 5, 2023.

Multimedia Appendix 2 Novel surveillance metrics for countries in sub-Saharan Africa for the week of May 5, 2023.

## Abbreviations

CDC: Centers for Disease Control
GDP: gross domestic product
GISAID: Global Initiative on Sharing All Influenza Data
PPE: personal protective equipment
SSA: sub-Saharan Africa
VOC: variant of concern
WHO: World Health Organization

## Data Availability

All data in this study are publicly available. COVID-19 transmission and death rates have been sourced from Our World in Data [43]. Information on sequenced SARS-CoV-2 specimens has been sourced from the Global Initiative on Sharing All Influenza Data (GISAID) [47].
