# Supplementary material for: Updated Surveillance Metrics and History of the COVID-19 Pandemic (2020-2023) in Sub-Saharan Africa: Longitudinal Trend Analysis
Source: JMIR Public Health Surveill. 2024 Oct 23;10:e53409. doi: 10.2196/53409 (PMC11541149; doi:10.2196/53409)
Supplement: Multimedia Appendix 1 [file publichealth_v10i1e53409_app1.docx]

**Multimedia Appendix 1.** Traditional COVID-19 surveillance metrics for countries in sub-Saharan Africa for the week of May 5, 2023.

| Country | New COVID-19 cases, n | Cumulative COVID-19 cases, n | 7-day moving average of new cases | Infection rate per 100,000 individuals | New deaths, n | Cumulative deaths, n | 7-day moving average of deaths | Death rate per 100,000 individuals | Conditional death rate |
| --- | --- | --- | --- | --- | --- | --- | --- | --- | --- |
| Angola | 0 | 105,384 | 0 | 0 | 0 | 1934 | 0 | 0 | 0.02 |
| Benin | 0 | 28,014 | 0 | 0 | 0 | 163 | 0 | 0 | 0.01 |
| Botswana | 2 | 329,862 | 1.29 | 0.47 | 0 | 2797 | 0.14 | 0 | 0.01 |
| Burkina Faso | 0 | 22,056 | 0 | 0 | 0 | 396 | 0 | 0 | 0.02 |
| Burundi | 0 | 53,749 | 1.29 | 0 | 0 | 15 | 0 | 0 | 0 |
| Cabo Verde | 0 | 63,702 | 14.71 | 0 | 0 | 414 | 0.14 | 0 | 0.01 |
| Cameroon | 0 | 125,036 | 0 | 0 | 0 | 1972 | 0 | 0 | 0.02 |
| Central African Republic | 0 | 15,367 | 0 | 0 | 0 | 113 | 0 | 0 | 0.01 |
| Chad | 0 | 7698 | 0 | 0 | 0 | 194 | 0 | 0 | 0.03 |
| Comoros | 0 | 9109 | 0 | 0 | 0 | 160 | 0 | 0 | 0.02 |
| Côte d'Ivoire | 1 | 88,329 | 0.43 | 0 | 0 | 834 | 0 | 0 | 0.01 |
| Democratic Republic of Congo | 0 | 96,652 | 14.71 | 0 | 0 | 1467 | 0.29 | 0 | 0.02 |
| Equatorial Guinea | 0 | 17,130 | 0 | 0 | 0 | 183 | 0 | 0 | 0.01 |
| Ethiopia | 5 | 500,867 | 2.86 | 0 | 0 | 7574 | 0 | 0 | 0.02 |
| Gabon | 0 | 48,992 | 0 | 0 | 0 | 307 | 0 | 0 | 0.01 |
| Gambia | 0 | 12,626 | 0 | 0 | 0 | 372 | 0 | 0 | 0.03 |
| Ghana | 0 | 171,653 | 0 | 0 | 0 | 1462 | 0 | 0 | 0.01 |
| Guinea | 0 | 38,563 | 0 | 0 | 0 | 468 | 0 | 0 | 0.01 |
| Guinea-Bissau | 0 | 9614 | 0 | 0 | 0 | 177 | 0 | 0 | 0.02 |
| Kenya | 0 | 343,074 | 0 | 0 | 0 | 5688 | 0 | 0 | 0.02 |
| Liberia | 0 | 8090 | 0 | 0 | 0 | 294 | 0 | 0 | 0.04 |
| Madagascar | 2 | 68,253 | 2 | 0.04 | 0 | 1424 | 0 | 0 | 0.02 |
| Malawi | 0 | 88,638 | 0.57 | 0 | 0 | 2686 | 0 | 0 | 0.03 |
| Mali | 0 | 33,145 | 0.14 | 0 | 0 | 743 | 0 | 0 | 0.02 |
| Mauritania | 0 | 63,663 | 1.29 | 0 | 0 | 997 | 0 | 0 | 0.02 |
| Mauritius | 197 | 304,233 | 213.86 | 15.56 | 0 | 1050 | 0.29 | 0 | 0 |
| Mozambique | 0 | 233,417 | 0 | 0 | 0 | 2243 | 0 | 0 | 0.01 |
| Namibia | 0 | 171,310 | 0 | 0 | 0 | 4091 | 0 | 0 | 0.02 |
| Niger | 0 | 9513 | 0 | 0 | 0 | 315 | 0 | 0 | 0.03 |
| Nigeria | 0 | 266,675 | 0 | 0 | 0 | 3155 | 0 | 0 | 0.01 |
| Republic of the Congo | 0 | 25,194 | 0 | 0 | 0 | 389 | 0 | 0 | 0.02 |
| Rwanda | 0 | 133,194 | 0 | 0 | 0 | 1468 | 0 | 0 | 0.01 |
| São Tomé and Príncipe | 0 | 6575 | 0.29 | 0 | 0 | 80 | 0 | 0 | 0.01 |
| Senegal | 0 | 88,997 | 0 | 0 | 0 | 1971 | 0 | 0 | 0.02 |
| Seychelles | 0 | 50,937 | 0 | 0 | 0 | 172 | 0 | 0 | 0 |
| Sierra Leone | 0 | 7762 | 0 | 0 | 0 | 125 | 0 | 0 | 0.02 |
| Somalia | 0 | 27,334 | 0 | 0 | 0 | 1361 | 0 | 0 | 0.05 |
| South Africa | 0 | 4,072,533 | 0 | 0 | 0 | 102,595 | 0 | 0 | 0.03 |
| Sudan | 0 | 63,993 | 0 | 0 | 0 | 5046 | 0 | 0 | 0.08 |
| Swaziland | 0 | 74,670 | 0 | 0 | 0 | 1425 | 0 | 0 | 0.02 |
| Tanzania | 0 | 43,078 | 0 | 0 | 0 | 846 | 0 | 0 | 0.02 |
| Togo | 0 | 39,491 | 0.57 | 0 | 0 | 290 | 0 | 0 | 0.01 |
| Uganda | 0 | 170,775 | 4.14 | 0 | 0 | 3632 | 0 | 0 | 0.02 |
| Zambia | 12 | 343,995 | 0 | 0.42 | 0 | 4058 | 0 | 0 | 0.01 |
| Zimbabwe | 2 | 264,816 | 1.71 | 0.10 | 0 | 5689 | 0.29 | 0 | 0.02 |
