# Supplementary material for: Updated Surveillance Metrics and History of the COVID-19 Pandemic (2020-2023) in Sub-Saharan Africa: Longitudinal Trend Analysis
Source: JMIR Public Health Surveill. 2024 Oct 23;10:e53409. doi: 10.2196/53409 (PMC11541149; doi:10.2196/53409)
Supplement: Multimedia Appendix 2 [file publichealth_v10i1e53409_app2.docx]

**Multimedia Appendix 2.** Novel surveillance metrics for countries in sub-Saharan Africa for the week of May 5, 2023.

| Country | Speed | Acceleration | Jerk | 7-day persistence effect on speed |
| --- | --- | --- | --- | --- |
| Angola | 0 | 0 | 0 | 0 |
| Benin | 0 | 0 | 0 | 0 |
| Botswana | 0.32 | 0.04 | 0.01 | 0.17 |
| Burkina Faso | 0 | 0 | 0 | 0 |
| Burundi | 0.01 | 0 | 0 | 0.01 |
| Cabo Verde | 2.48 | -0.60 | -0.24 | 3.38 |
| Cameroon | 0 | 0 | 0 | 0.02 |
| Central African Republic | 0 | 0 | 0 | 0 |
| Chad | 0 | 0 | 0 | 0 |
| Comoros | 0 | 0 | 0 | 0 |
| Côte d'Ivoire | 0 | 0 | 0 | 0 |
| Democratic Republic of Congo | 0.10 | -0.03 | 0 | 0.21 |
| Equatorial Guinea | 0 | 0 | 0 | 0 |
| Ethiopia | 0 | 0 | 0 | 0 |
| Gabon | 0 | 0 | 0 | 0 |
| Gambia | 0 | 0 | 0 | 0 |
| Ghana | 0 | 0 | 0 | 0 |
| Guinea | 0 | 0 | 0 | 0 |
| Guinea-Bissau | 0 | 0 | 0 | 0 |
| Kenya | 0 | 0 | 0 | 0 |
| Liberia | 0 | 0 | 0 | 0 |
| Madagascar | 0.05 | 0 | 0 | 0.08 |
| Malawi | 0 | 0 | 0 | 0.01 |
| Mali | 0 | 0 | 0 | 0 |
| Mauritania | 0.03 | 0 | 0 | 0.12 |
| Mauritius | 16.91 | 0 | 0 | 18.67 |
| Mozambique | 0 | 0 | 0 | 0 |
| Namibia | 0 | 0 | 0 | 0 |
| Niger | 0 | 0 | 0 | 0 |
| Nigeria | 0 | 0 | 0 | 0 |
| Republic of the Congo | 0 | 0 | 0 | 0 |
| Rwanda | 0 | 0 | 0 | 0 |
| São Tomé and Príncipe | 0.38 | 0 | 0 | 0.07 |
| Senegal | 0 | 0 | 0 | 0 |
| Seychelles | 0 | 0 | 0 | 0 |
| Sierra Leone | 0 | 0 | 0 | 0 |
| Somalia | 0 | 0 | 0 | 0 |
| South Africa | 0 | 0 | 0 | 0 |
| Sudan | 0 | 0 | 0 | 0 |
| Swaziland | 0 | 0 | 0 | 0.54 |
| Tanzania | 0 | 0 | 0 | 0.01 |
| Togo | 0.01 | 0 | 0 | 0.01 |
| Uganda | 0.03 | 0 | 0 | 0.02 |
| Zambia | 0 | 0 | 0 | -0.18 |
